# A woman with eptifibatide (integrilin)‐induced thrombocytopenia following treatment of a clot in her coronary artery: A case report and literature review

**DOI:** 10.1002/ccr3.8694

**Published:** 2024-03-28

**Authors:** Morteza Safi, Roozbeh Nazari, Nahid Senobari, Homa Taheri, Pouya Ebrahimi

**Affiliations:** ^1^ Cardiovascular Research Center Shahid Beheshti University of Medical Sciences, Modarres Hospital Tehran Iran; ^2^ Cedars‐Sinai Cardiology Department California USA; ^3^ Tehran Heart Center Cardiovascular Disease Research Institute, Tehran University of Medical Sciences Tehran Iran

**Keywords:** acute coronary syndrome, adverse drug event, eptifibatide, thrombocytopenia, thrombosis

## Abstract

Eptifibatide, a GPIIb/IIIa receptor inhibitor, has shown its efficacy and safety in patients with high clot burden in their coronary vessels. It is widely used in patients with this condition. However, this medication use is accompanied by complications in some cases. Thrombocytopenia which is a relatively common condition in patients admitted to the hospital, especially in the acute setting, can be caused by medications. This condition can occur as an antibody or non‐antibody‐mediated process, caused by medications, such as heparin, clopidogrel, and eptifibatide. In this case, we present a woman with acute coronary syndrome and a complex lesion with a clot in her coronary vessel who was treated with eptifibatide. It led to asymptomatic thrombocytopenia. Once detected in laboratory data, the infusion was held, and the platelet count recovered in less than 5 days without additional treatment for this adverse effect. Eptifibatide is a medication used to treat acute coronary syndrome patients with a large thrombus in their coronary vessels. The mechanism of inducing thrombocytopenia by eptifibatide has not been proven yet, but it might be related to IgG antibodies. The severity of the disease can vary significantly, and the treatment is based on this factor. However, the main pillar of the treatment is the cessation of eptifibatide as soon as possible. This case draws the attention of physicians to one of the infrequent adverse effects of a commonly used medication in cardiology patients. Thrombocytopenia and its manifestations should be investigated and considered in patients who receive eptifibatide.

## 1 INTRODUCTION

Eptifibatide (integrilin) and Tirofiban are two medications used in patients with acute coronary syndrome to prevent platelet aggregation and clot formation. The mechanism of action is inhibition of the GPIIb/IIIa receptor and consequently, fibrinogen binding inhibition^1^, ^2^ The intravenous and intracoronary infusion of this group of medications has shown their efficacy in patients with a high burden of thrombus in their coronary vessels.^3^ Taking advantage of these medications was correlated with a significantly improved cardiovascular and overall outcome in patients with a high burden of thrombus.^3^, ^4^ Sharma. et al. conducted a clinical trial and showed that intra‐coronary administration of tirofiban was associated with improved clinical and angiographic outcomes in patients with high thrombus burden prior to their percutaneous coronary intervention (PCI).^4^

Eptifibatide‐induced thrombocytopenia is a rare condition caused by IgG or non‐antibody‐related causes reported in patients without previous exposure to this medication and has a prevalence of 0.1%–0.2%.^1^, ^2^, ^3^ Mostly, this adverse effect of this medication happens in the first 24 h after the initiation of the infusion of the medication.^5^ Eptifibatide thrombocytopenia can cause severe complications such as bleeding, paradoxical thrombosis, and prolonged hospital stays. Therefore, their use should be limited to selected cases with the necessary need for this medication, such as considerably diminished blood flow by a large thrombus.^5^

This study presents a case of a woman who presented to the emergency room with chest pain and received eptifibatide due to the observation of a large clot in her left circumflex artery (LCX). This woman had a declining platelet count trend with the start of the infusion and a reversal of the trend with the cessation of the medication.

## 2 CASE PRESENTATION

### 2.1 Case history and examination

The patient was a 58‐year‐old woman who presented with retrosternal pain that started 2 weeks before coming to the emergency department (ED). She described the pain as burning, intermittent (continued for 20 min), and improving following nitroglycerin use or resting. The pain occurred twice during the last 2 weeks before her presentation. The patient had gone to the emergency room 1 week before due to her previous chest pain episode. She was advised to be admitted to the hospital for more evaluation by a coronary angiography (CAG) by the medical team. However, she had declined the medical recommendation due to her occupational condition. She had no past medical history of ischemic heart disease but had hypertension and dyslipidemia from several years ago. In her past drug history, nitroglycerin, metoprolol, atorvastatin, and valsartan were mentioned. In her initial assessment, her vital signs were normal, and her physical examination was unremarkable.

### 2.2 Methods

**FIGURE 1:**
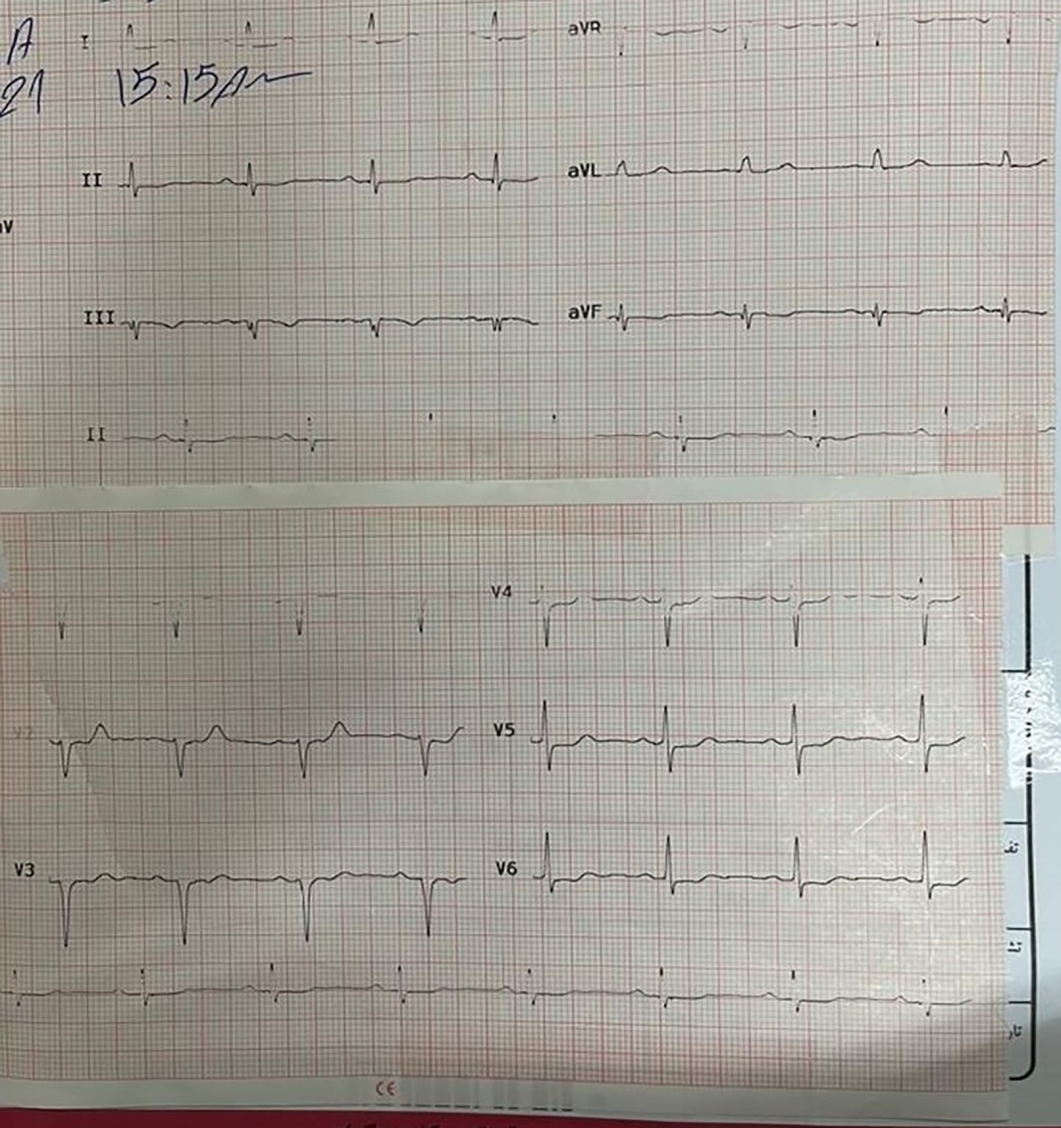
ST‐segment depression was detected in several precordial leads of the patient's ECG.

**FIGURE 2:**
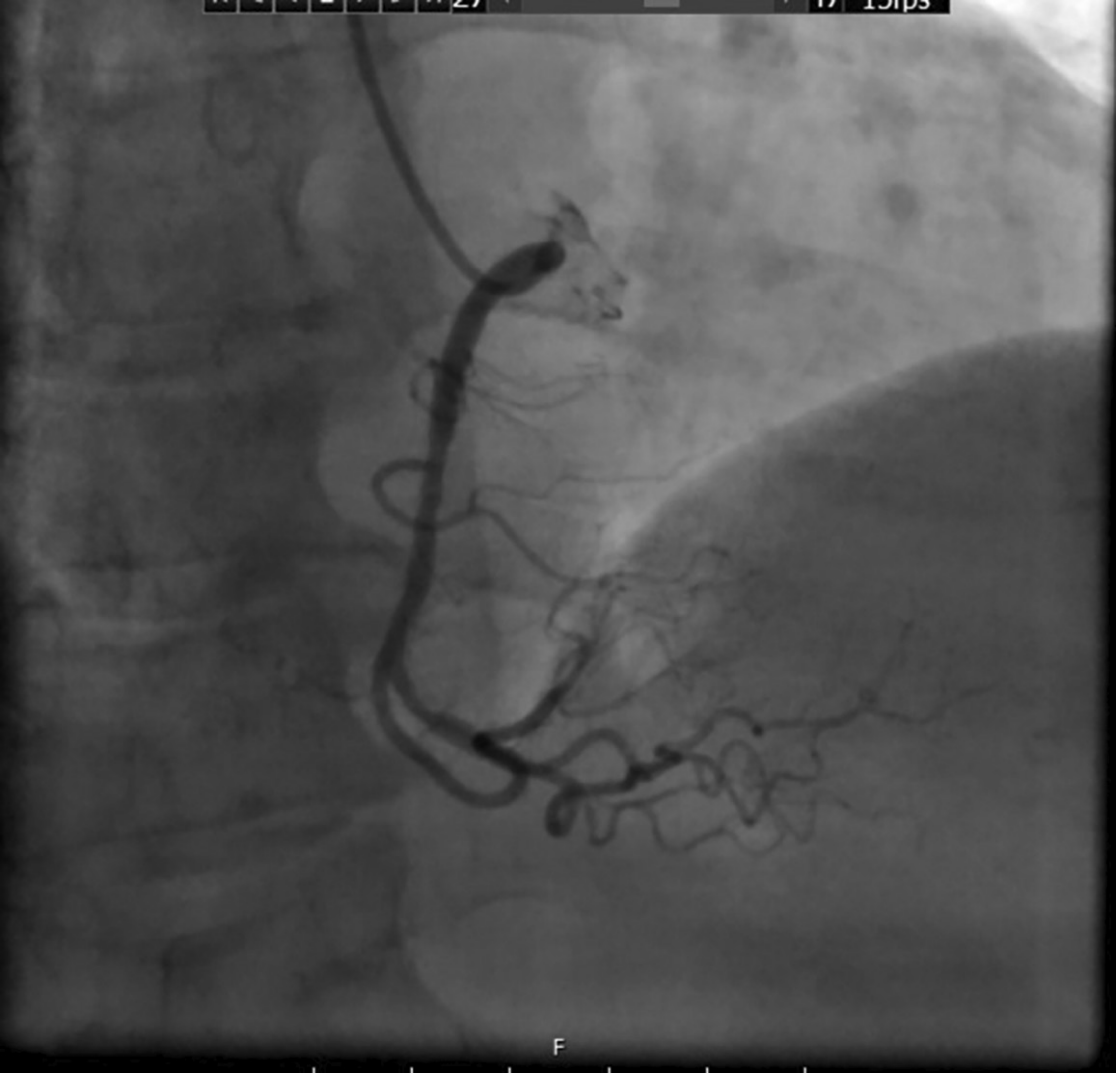
There was no significant lesion in the patient's right coronary artery.

**FIGURE 3:**
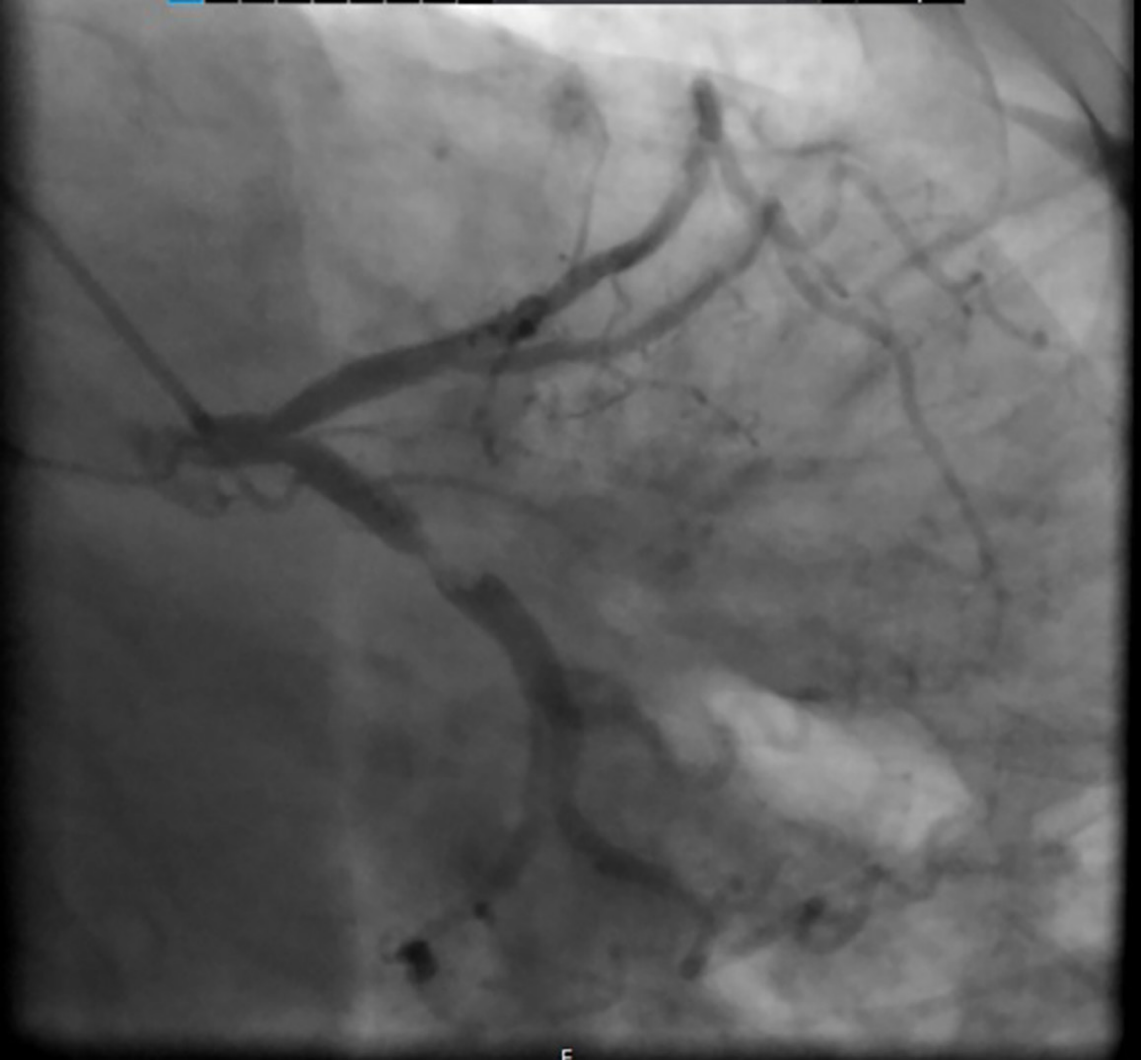
LCX was the dominant artery, there was a complex lesion with a clot in this artery.

**FIGURE 4:**
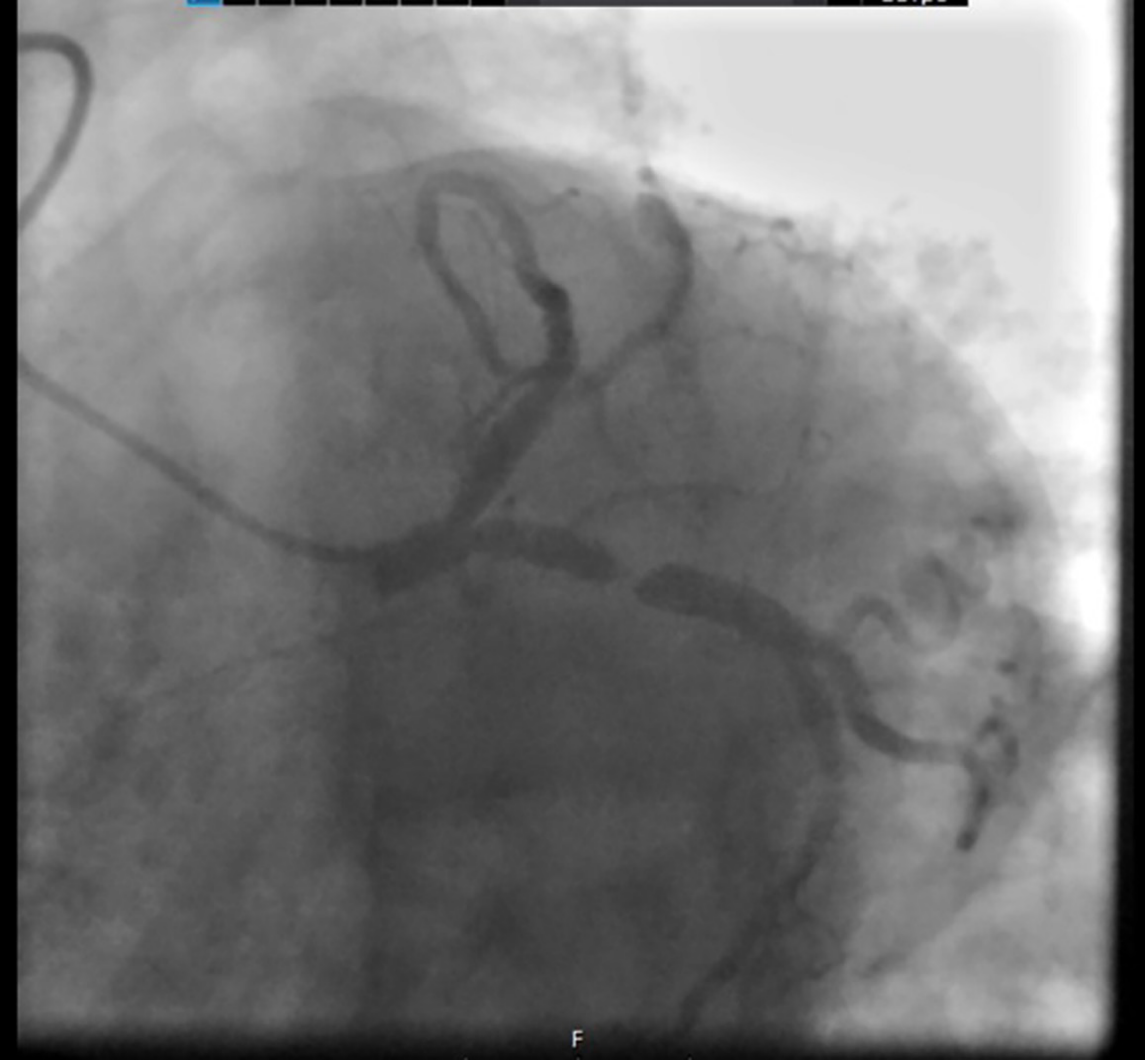
LCX was the dominant artery and there was a complex lesion with a clot in this artery.

Following obtaining an electrocardiogram (ECG), which was immediately after taking the patient's history and performing a physical exam, ST‐segment depression was detected in several precordial leads (Figure 1). At this presentation, all laboratory data, chest x‐rays, and troponin‐level results were unremarkable. Considering her typical chest pain for ischemic heart disease and ECG evidence, she was admitted to the coronary care unit, and CAG was planned to be done urgently after her preparation. An urgent echocardiography while the catheter laboratory was being prepared demonstrated a left ventricular ejection fraction of 55%, and an abnormal septal motion. Then the patient was urgently transferred to the catheter laboratory. The CAG revealed that her right coronary artery had no significant lesion (Figure 2), her left anterior descending artery had an intimal irregularity in its proximal part, and her LCX had a complex lesion with a clot and was dominant (Figures 3 and 4). Due to clot observation, anticoagulation therapy was planned, and an integrin alpha IIbbeta3 antagonist eptifibatide drip infusion was initiated immediately.

The patient's condition remained stable, and no abnormal sign or symptom was seen until 3 h after the initiation of the medication. The patient stated feeling unusual fatigue. The cardiologist immediately evaluated the vital signs performed a complete physical exam and found no remarkable signs. Therefore, to make sure about the patient's appropriate clinical condition a series of laboratory data was requested. The complete blood counts 3 h after the initiation of the integrin alpha IIbbeta3 antagonist eptifibatide showed a decline of platelets from 206,000 cells/mm^3^ to 80,0000 cells/mm^3^. Since the other medications that the patient was receiving were administered to her in her previous chest pain episode in the emergency room or were not known as a potential cause by eptifibatide. Moreover, a heparin‐induced thrombocytopenia test was done and was negative. Suspecting eptifibatide‐induced thrombocytopenia, the drip infusion of the medication is stopped immediately. However, other treatments for acute coronary syndrome have been continued. Notably, it was concluded that the patient's fatigue was not due to her thrombocytopenia since her condition improved after a short time of resting and before the initiation of any change in her treatment. The patient's history was evaluated completely and no previous drug reaction or any exposure to this group of medication was found. The observed drug reaction was added to the patient's file as a note for her future visits or admissions.

### 2.3 Conclusion and results

**FIGURE 5:**
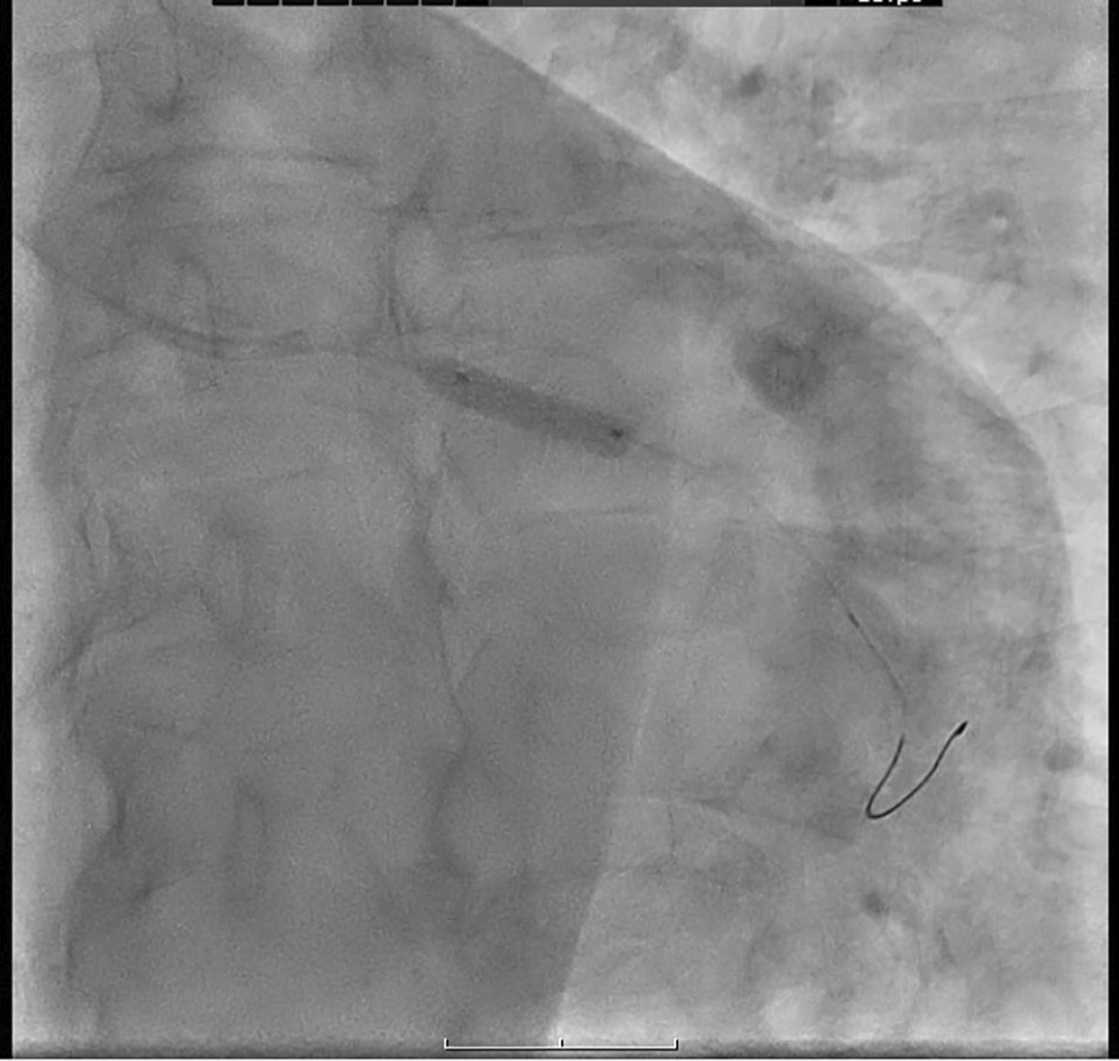
Second percutaneous coronary angiography and stent implantation in the LCX artery.

**FIGURE 6:**
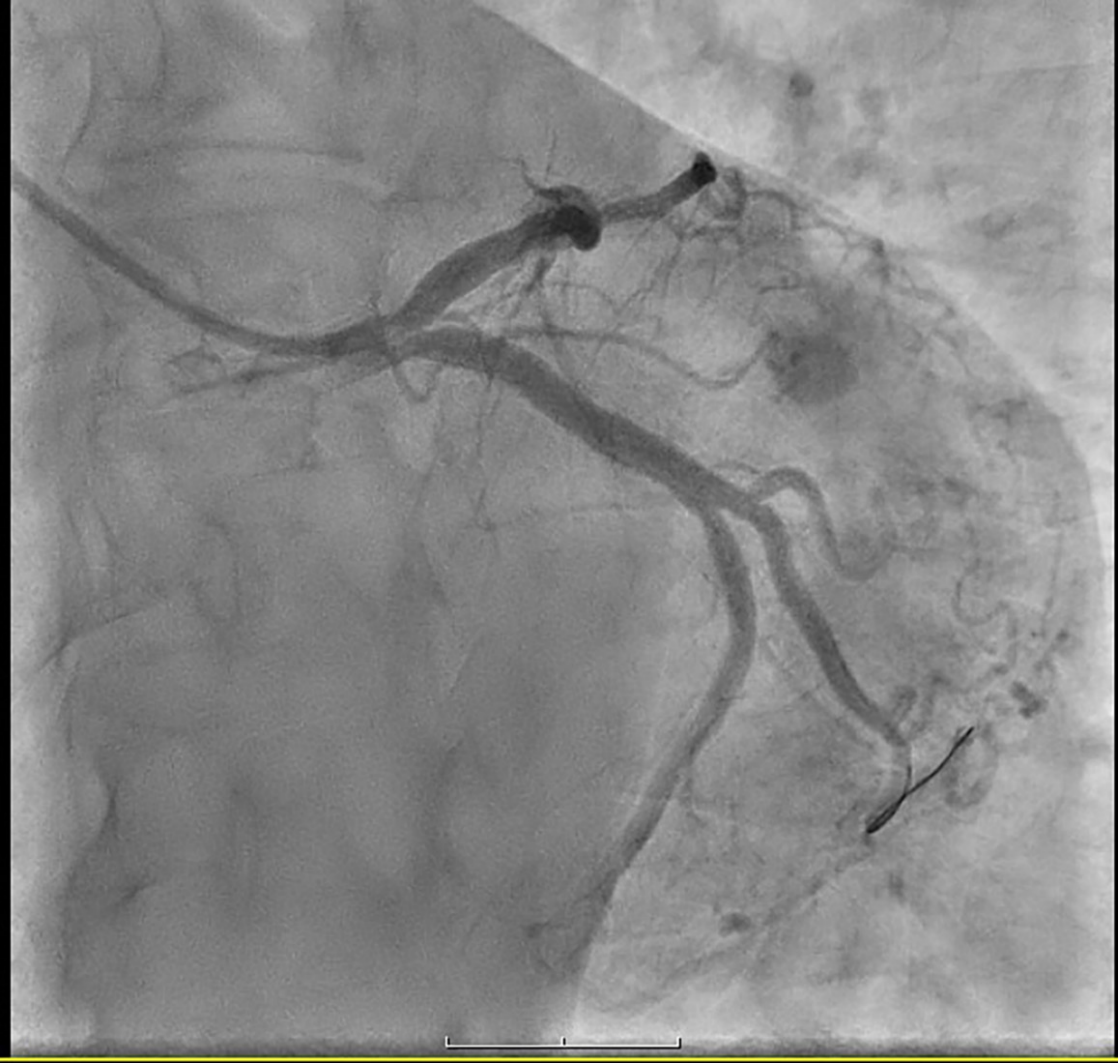
Second percutaneous coronary angiography, after stent implantation in the LCX artery.

**FIGURE 7:**
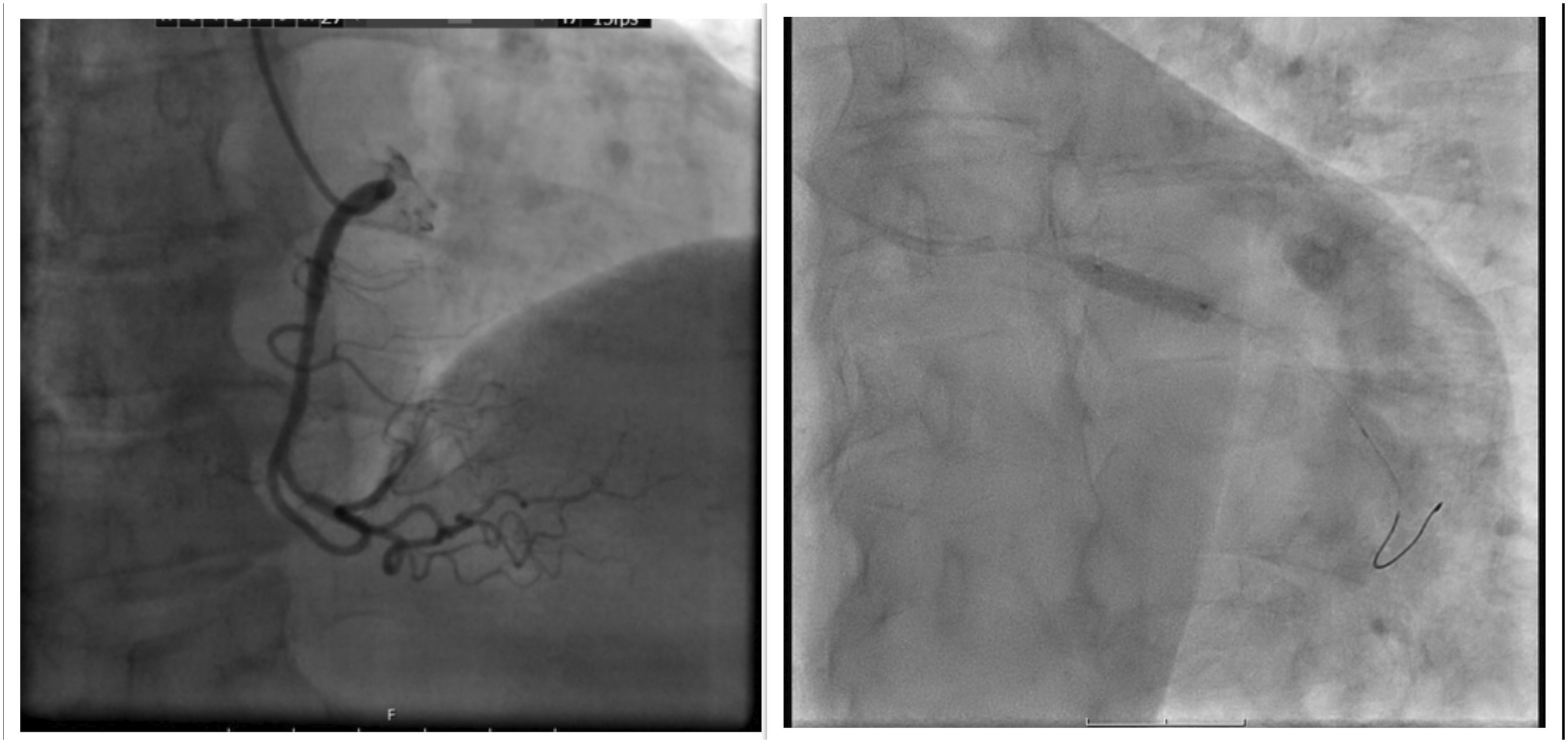
On the left: In the first percutaneous coronary intervention (PCI) there was no significant lesion in the patient's right coronary artery. On the right: Second percutaneous coronary angiography and stent implantation in the LCX artery.

In the next complete blood count after cessation of infusion which was performed about 14 h after the change in the medication, the platelet count increased to 90,000, and then 101,000, 120,000, 143,000, 189,000, 205,000, and 212,000 cells/mm^3^ in the following 5 days (Table 1). Clexane, which was the medication that the patient had received in her last emergency room admission due to chest pain showed no adverse effect was initiated instead of eptifibatide. One week after the admission, the CAG was repeated, and percutaneous catheter intervention was done on the LCX by stent implantation in this artery (Figures 5 and 6). The patient's condition improved, and she was discharged 1 day later without any complications. Treatment with aspirin, clopidogrel, metoprolol, atorvastatin, valsartan, and as‐needed nitroglycerin was continued. In the 1‐, 2‐, and 8‐week follow‐up visits, the patient's symptoms were resolved, and her general condition improved significantly. No abnormal sign or symptom was detected in the patient's follow‐up visits. Figure 7 provides both the first and the second CAG of the patient.

**TABLE 1:** After holding the infusion of eptifibatide, the platelet count increased to 90,000 and gradually up to 205,000 cells/mm^3^ in 5 days.

Date	Platelet count	Normal range
November 13, 2023‐03:06 a.m.	85,000	105,000–450,000
November 13, 2023‐04:45 p.m.	90,000	105,000‐450,000
November 13, 2023‐10:55 p.m.	101,000	105,000‐450,000
November 14, 2023‐10:09 p.m.	120,000	105,000‐450,000
November 15, 2023‐10:43 p.m.	143,000	105,000‐450,000
November 16, 2023‐10:28 p.m.	189,000	105,000‐450,000
November 17, 2023‐10:35 p.m.	205,000	105,000‐450,000

## 3 DISCUSSION

Thrombocytopenia is defined as a platelet count of less than 150,000 cells/mm^3^. It is divided into three types: mild (100,000–149,000), moderate (50,000–99,000), and severe, which means less than 50,000 cells/mm^3^ ^6^ Thrombocytopenia is relatively common in hospitals, especially acute settings, with a prevalence ranging from 15% to 40%.^7^ The first step in approaching thrombocytopenia is repeating the complete blood count to make sure it is not a laboratory error, pseudo‐thrombocytopenia, or thrombocytopenia due to hemodilution.^7^ Heparin is one of the medications that can cause thrombocytopenia by causing anti‐body generation to form platelet factor 4 (PF4) and the heparin complex. Clopidogrel is another drug that can cause thrombocytopenia by inducing thrombotic thrombocytopenic purpura (TTP) or allergic reactions.^8^, ^9^ The list of drug‐induced thrombocytopenia is not limited to these medications. To differentiate the cause of thrombocytopenia these points should be considered that the thrombocytopenia caused by eptifibatide is steeper and occurs early (in hours) after initiation. However, heparin‐induced thrombocytopenia is milder most of the time and it occurs in the fourth to twentieth day after initiation of medication.^10^ Moreover, another characteristic of eptifibatide‐induced thrombocytopenia is its response to the cessation of medication. Similar to our patient, only cessation of this medication infusion would lead to recovery of the platelet count in most cases.^10^

Eptifibatide is a medication used for patients with large thrombus in their coronary vessels, which has caused acute coronary syndrome. This drug inhibits GPIIb/IIIa and prevents platelet aggregation and clot formation with a mechanism separate from cyclooxygenase inhibitors and P2Y12 inhibitors. This would increase the rate of successful PCI and could cause better outcomes in patients undergoing this process. This medication is mainly used for non‐ST‐elevated acute coronary syndrome to improve interventional success.^11^ The platelet anti‐aggregation effect of the bolus and drip infusion of eptifibatide can be observed as soon as 1 min and lasts for 4–6 h.^12^

The mechanism of eptifibatide‐induced thrombocytopenia is not well known, but it has been attributed to both antibody‐mediated and non‐antibody‐mediated mechanisms.^13^, ^14^ There are studies stating the probable mechanism of eptifibatide‐induced thrombocytopenia. Bougie. et al. observed the tendency of attachment between the antibodies that recognize multiple target epitopes on the GPIIb/IIIa and the drug in a trial conducted on nine patients with tirofiban and eptifibatide‐induced thrombocytopenia. They stated that this process can be seen as a natural process or as a cause of prior exposure to the medication.^13^ In another study, Gao et al. observed thrombocytopenia caused by IG‐g‐induced platelet aggregation in the presence of eptifibatide when they incubated normal human platelets with serum containing eptifibatide‐dependent antibodies. They concluded that this might be a mechanism of eptifibatide‐induced thrombocytopenia.^15^

Most of the manifestations of this kind of thrombocytopenia are negligible, such as petechia or minor bleeding from intravenous access. However, more serious cases, such as gastrointestinal bleeding, hematuria, and life‐threatening bleeding that need immediate management can be seen^16^ The management of eptifibatide thrombocytopenia depends on the severity of the condition and the platelet count. Similar to other suspected drug‐induced thrombocytopenia other causes should be investigated and ruled out by a complete history taking, physical examination, and laboratory data evaluation. Then the causing medication should be stopped as soon as possible and based on the severity of thrombocytopenia, observation, intravenous immunoglobulin (IVIG), high‐dose corticosteroids, or even platelet transfusion in severe cases.^17^ Most of the patients are responsive to the cessation of medication, and the platelet count recovers mostly in 1–5 days. On the other hand, cases with a platelet count of less than 10,000 might need platelet infusion and cessation of other anti‐platelet drugs. Although using IVIG as a treatment for this condition is not an option, corticosteroids might be used in refractory cases with severe and hard‐to‐stop bleeding.^18^, ^19^ In Table 2, similar cases of eptifibatide‐induced thrombocytopenia are mentioned.

**TABLE 2:** A literature review of similar cases of eptifibatide‐induced thrombocytopenia.

Title and Authors	Age and sex	Diagnosis (reason for receiving eptifibatide)	Platelet count trend and symptoms	Treatment and the final consequence
Eptifibatide‐induced severe thrombocytopenia after ST‐elevation myocardial infarction (STEMI): A case report Zaid Gheith, et al.^16^	69‐year‐old male	ST‐elevation myocardial infarction	Gross hematuria and a petechial rash, platelet count of 3000cells/mm^3^	Holding of eptifibatide, transfusion of 1 unit of platelets raised platelet count to 14,000/μL. Trend up slowly to 35,000 in 2 days, resolution of the patient's hematuria, discharged after 72 h
Acute profound thrombocytopenia induced by eptifibatide causing diffuse alveolar hemorrhage Gregory Byrd, et al.^14^	50‐year‐old male	Inferior‐posterior STEMI	Severe cough and mild–moderate hemoptysis, platelets declined from 370,000cells/mm^3^ to 5000cells/mm^3^ within 12 h	Received 1 unit of platelets, the ticagrelor and eptifibatide were discontinued, and platelets began to rebound. Without further treatment, the patient was discharged with a normal platelet count 9 days later
A rare case of eptifibatide‐induced thrombocytopenia Khalil Kamar, et al.^20^	76‐year‐old female	Non‐ST elevation myocardial infarction (NSTEMI)	On Day 7, after eptifibatide infusion initiation, the platelet count dropped to 3000 from 243,000 (normal 130–400 K/uL)	Cessation of eptifibatide, a continuation of aspirin and clopidogrel, one unit of platelets transfused On Day 7: The platelet count increased to 78 K/uL and the trend was rising to normal count
Delayed‐onset eptifibatide‐induced thrombocytopenia Travis Huffman, et al.^5^	62‐year‐old male	Significant thrombus burden in coronary arteries (Acute Coronary Syndrome)	5 days after initiation of eptifibatide, severe thrombocytopenia developed. platelet count dropping from 249,000/μL on admission to less than 1000/μL	Discontinuation of eptifibatide and heparin switched to argatroban, recovery of platelet counts to 38,000/μL over the next 2 days
Eptifibatide‐induced acute profound thrombocytopenia: a case report Christos Graidis, et al.^21^	44‐year‐old male	Acute coronary syndrome	Four hours post‐eptifibatide initiation, profound thrombocytopenia development, platelet count dropping over 90% from baseline to 15,000/mm^3^	Cessation of all antiplatelet products including heparin, eptifibatide dual antiplatelet therapy (DAPT) for 48 h, recovery of platelet counts to normal level
Eptifibatide‐induced thrombocytopenia Marwan Refaat, et al.^22^	34‐year‐old male	Severe diffuse epicardial CAD, proximal occlusion of the left circumflex (LCX) artery	Acute onset nausea, diaphoresis, hypotension, chest discomfort, and increased work of breathing with no changes on the electrocardiogram 5 days after initiation of eptifibatide. Profound thrombocytopenia platelet counts of 6000, down from the Baseline of 298,000cells/mm^3^	Immediate cessation of ASA, clopidogrel, subcutaneous heparin, and eptifibatide, Recovery of platelet count without any transfusion, aspirin, subcutaneous unfractionated heparin, and clopidogrel that were restarted the following day was continued, discharged the day later
Eptifibatide‐induced thrombocytopenia: with thrombosis and disseminated intravascular coagulation immediately after left main coronary artery percutaneous coronary angioplasty Michael W Tempelhof, et al.^23^	73‐year‐old man	A lesion in the left main coronary artery/minimal area of vascular lumen/significant stenosis	Baseline CBC 238,000 cells/mm^3^, 3 h after cardiac catheterization/CBC revealed significant thrombocytopenia: a platelet count of 78,000 and 64,000 1 h later. Six hours after his PCI/became lightheaded/an abnormal bowel movement. Systolic blood pressure was 80 mmHg heart rate was 50 beats/min. Evidence of pulmonary emboli and disseminated intravascular coagulopathy (DIC)	Improvement of symptoms and blood pressure improved after the administration of 1.5 L of normal saline solution. Argatroban initiation intubation, hemodialysis, and infusion of packed red blood cells, platelets, fresh frozen plasma, and cryoprecipitate. Gradual platelet recovery. Discharged in good condition 19 days later
Eptifibatide‐induced thrombocytopenia leading to acute stent thrombosis Dezsi.et al.^24^	71‐year‐old female	ST‐elevation myocardial infarction	A severe drop in platelet counts from 210,000/μL to 35,000/μL after the initiation of eptifibatide, 1 h later, still under eptifibatide effect and with severe thrombocytopenia, acute stent thrombosis developed	Cessation of eptifibatide infusion, due to paradoxical high platelet reactivity at the time of stent thrombosis decreased radically with eptifibatide washout

Abbreviation: PCI, percutaneous coronary intervention.

In our case, fortunately, the patient had only laboratory manifestations (moderate thrombocytopenia), and there was no clinical presentation such as minor or major bleeding. Since the patient had received clexane before and showed no adverse effect to this medication, eptifibatide was replaced by this medication. The other positive aspect of this case was being responsive to the cessation of drip infusion and a recovering platelet count in less than 24 h without any additional treatment.

## 4 CONCLUSION AND CLINICAL LEARNING POINT

In this case, we presented a woman without any history of thrombocytopenia or allergy to medications with a large clot in her LCX, which had been decided to be managed with eptifibatide. However, the moderately detected thrombocytopenia caused a change in the treatment strategy and the cessation of this medication. It can be concluded that in the use of eptifibatide, this adverse, although uncommon, should be considered by the physician, and if it occurs, should be approached by the cessation of medication and also treated based on the severity of thrombocytopenia and the clinical response of the patients. Moreover, during the transfusion, the platelet count should be monitored carefully, and regular evaluation for any abnormal signs and symptoms such as petechia, any minor or major bleeding, and epistaxis should be performed. Most cases are self‐limited and will be managed by cessation of the medication. However, in severe cases, IVIG, high‐dose corticosteroids, or even platelet transfusion might be indicated.

## 5 STUDY LIMITATION

Since the echocardiography of the patient was not recorded as a video in the center, we could not access it and it was the limitation of our study. However, the written report of the echocardiography was documented and accessible, which is included in the case presentation.

## AUTHOR CONTRIBUTIONS

**Morteza Safi:** Conceptualization; data curation; formal analysis; project administration; supervision. **Roozbeh Nazari:** Conceptualization; data curation; resources; software; validation. **Nahid Senobari:** Investigation; methodology; resources; validation; writing – original draft; writing – review and editing. **Homa Taheri:** Writing – original draft; writing – review and editing. **Pouya Ebrahimi:** Methodology; supervision; visualization; writing – original draft; writing – review and editing.

## FUNDING INFORMATION

The authors declared that this study has not received financial support from any organization.

## CONFLICT OF INTEREST STATEMENT

The authors declared no conflict of interest.

## CONSENT

Written informed consent was obtained from the patient to publish this report under the journal's patient consent policy.

## Data Availability

For more information and data, the corresponding author should be contacted. The data will be available for reasonable requests.
